# Progress in Surgery

**Published:** 1902-10-11

**Authors:** 


### Progress in Surgery.

(*Continued from page* 17.)

### GENITO-URINARY SURGERY.

**Grafting the Divided Ureter.**—Brin[3] has discussed the treatment of cases of accidental or surgical division of the ureter. He successfully performed first a uretero-cutaneous graft and subsequently a uretero-vesical graft on the same patient. The cutaneous graft is serviceable if the patient is weak or the operation severe, but it leads to a grave infirmity. When the ureter is too short to graft on to the bladder it may be implanted in intestine without fear of ascending infection Brin thinks. Vesical grafting is the ideal procedure, though difficult to effect. The author split the layers of the bladder around the incision made to introduce the ureter, and sutured the end of the latter to the edge of the mucous membrane, and the wall of the ureter to the outer wall of the bladder. No hydronephrosis or other trouble ensued.

**Rupture of the Bladder.**—James Pedersen, New York,[4] records an example of successful treatment of this affection. The patient, a man of 42, fell from a window. Rupture of the bladder was suspected four hours later and a soft catheter passed. A small quantity of bloody urine was obtained. Four ounces of boric lotion were injected through the catheter. Three ounces returned. Six ounces were then injected and only four returned. Then a third injection was made. In all seven ounces of the lotion were retained. An operation was performed nine hours after the accident, a sound being passed into the bladder and cut down upon suprapubically. The point of the instrument was found to be immediately beneath the peritoneum. On opening the peritoneum a transverse rent in the bladder two and a half inches long was found just within the peritoneal reflexion. The operation had to be hurried and therefore the rent was closed with a continuous suture of catgut. This brought the edges closely together. A quantity of bloody fluid and clots were removed from the pelvis. Then the membranous urethra was incised and a catheter tied in. Two wick drains were passed through the lower part of the upper wound into the retro-vesical space Some leakage followed from the bladder wound and continued for ten days. The patient now urinates normally. D. N. Eisendrath, Chicago,[5] relates a case of extra-peritoneal rupture in a boy of five years of age, who had been run over by a street car. There was retention of urine, but a catheter only drew off a table-spoonful of bloody urine. Dulness extended from the umbilicus to the pubes, and tenderness. On supra-pubic incision, 26 hours after the injury, the prevesical space was found filled with bloody fluid, and the peritoneum pushed up to the level of the umbilicus with blood and urinous effusion. The pubic bones were separated at the symphysis. A punctured wound of the bladder was found opposite the projecting pubic bone. In addition the urethra was torn through at the junction of the membranous and bulbous portions, so that the bladder was practically floating about in the pelvis. The punctured wound was sutured after cystotomy; then perineal section was performed and retrograde catheterisation adopted to find the proximal end of the torn urethra. A catheter was passed and tied in. The supra-pubic wound gradually healed, the urethral wound gave trouble and a permanent alteration of the urethral curve resulted from the pelvic injury. We may here briefly describe Jonnesco's mode of suture of the bladder after suprapubic cystotomy described by Balacescu.[6] The bladder is prepared by internal administration of salol or urotropine for a few days and by washing out with 1 in 2,000 potassium permanganate solution or 1 in 5,000 silver nitrate solution. The peritoneum is separated for a short distance from the vertex and posterior surface of the bladder before opening it. To close the wound cut off from the left side of the bladder wound a strip of mucosa half a centimetre thick and extending the whole length of the wound, then sew the cut edge of the mucosa on the left side with the finest catgut to that of the right side, then suture the muscularis of the right side to the under side of the muscular flap on the left at its base. Finally, suture the free edge of the muscular flap to the surface of the bladder. In only one case out of twelve was there any leakage. In some the patients were allowed to micturate from the first without using a catheter either retained or at intervals.

**Partial Resection of the Bladder for Carcinoma.**—L. McGavin[7] reports a case of this operation. The patient was a woman of 72. For six months she had had from time to time painless hæmaturia, slight undue frequency, and tenderness over the pubes. A rough patch could be felt with a bladder sound. At the operation the urethra was first dilated and a digital examination made. A large warty growth, occupying an area of 2 by 1½ inches, was felt lying about an inch to the left of the left side of the trigone. It was movable on the deeper layers of the bladder. Suprapubic cystotomy was performed, and the growth snipped out with scissors. The entire thickness of the bladder wall down to the pelvic fascia was removed, and the edges of the hole could not be well approximated. For this reason the remainder of the tissue between the hole and the median incision in the bladder was excised. The difficulty of suturing the edges now left was overcome by stripping up the bladder and left ureteral orifice from the pelvic fascia and turning the left half of the bladder forwards towards the middle line. Twenty-five Lambert sutures were used to close the wound in the bladder. Thus the left ureteral orifice came to lie about half an inch from the line of suture. About one-fifth of the entire surface of the bladder was removed. Recovery was rapid. The author quotes Sir H. Thompson's advice to the effect that non-pedunculated growths, if the base is hard, should be left alone, because they cannot be separated from the deeper parts of the bladder wall, and any ablation of this would be fatal. Also, Jacobson says that for resection "the growth must be situated on the upper part of the bladder."

**Suprapubic Cystoscopy.**—Kraske[8] has twice recently examined the bladder through a suprapubic fistula established by ordinary high cystotomy. In both cases urethral cystoscopy was impracticable. A clear and extensive view of the inner surface of the bladder, especially of the fundus and the prostatic portion, is obtained. The author has devised means for systematically adopting this mode of examining the bladder when the latter is intact. A good view of the vesical orifice in prostatic hypertrophy, and of the configuration and degree of enlargement of the prostate, is obtained. As a preliminary to Bottini's operation, which the author considers the best for enlarged prostate, it is of special value. The Bottini operation can be performed by it under control of the eye. The simplest mode of performing suprapubic cystoscopy is by using a straight cystoscope with a pointed end like a trocar, which makes its own puncture. If a fistula exists it can be dilated by a laminaria tent, or the cystoscope can be passed through the cannula of a large trocar when the original puncture is made.

**Catheterisation of the Ureters and Radiography.** G. v. Illyés[9] states that the idea of making the course of the ureters visible by introducing a catheter which produced a radiographic shadow originated with Tuffier in 1899, and that the first accounts of such radiographs actually taken were given by Schmidt and Kolischer in Chicago and Loewenhardt in Breslau. Von Illyés relates four cases in which the method of investigation was used in Dollinger's Clinic in Buda-Pesth. The ureter-catheter was made opaque by passing into it a fine silver stylet. In case 1 it was questionable whether a tumour was a movable spleen or a movable kidney. The latter was excluded by the radiograph showing that the course of the ureter was normal. In case 2 the catheter was seen to have penetrated only as far as the sacro-iliac joint and to touch at that level something which gave a spherical shadow. This was considered to be an encysted stone, and subsequently an operation showed the diagnosis was correct. In case 3 the catheter was seen to have been arrested at the linea innominata, the arrest being due to a bend in the ureter of a movable kidney. In case 4 the catheter was arrested similarly by a kidney tumour, which had compressed the ureter. This mode of examination is thus seen to be capable of giving useful information. It is suggested that the catheter might be filled with an emulsion of subnitrate of bismuth to render it opaque.

[3] Vide Epitome No. 67, Brit. Med. Jour., Feb. 1, 1902. [4] Med. Rec., March 22, 1902, p. 475. [5] Annals of Surgery, February 1902, p. 288. [6] Vide Abst. Annals of Surgery, May 1902, p. 695. [7] Brit. Med. Jour., March 22, 1902, p. 711. [8] Centralb. f. Chir., Feb. 8, 1902, p. 153. [9] Vide Centralb. f. Chir., April 12, 1902, p. 426.

